# Collective Anomalies Detection for Sensing Series of Spacecraft Telemetry with the Fusion of Probability Prediction and Markov Chain Model

**DOI:** 10.3390/s19030722

**Published:** 2019-02-11

**Authors:** Jingyue Pang, Datong Liu, Yu Peng, Xiyuan Peng

**Affiliations:** School of Electronics and Information Engineering, Harbin Institute of Technology, Harbin 150080, China; jypang@hit.edu.cn (J.P.); pengyu@hit.edu.cn (Y.P.)

**Keywords:** telemetry series, collective anomalies, Markov chain, probability prediction, false positive, Gaussian process regression, relevance vector machine

## Abstract

Telemetry series, generally acquired from sensors, are the only basis for the ground management system to judge the working performance and health status of orbiting spacecraft. In particular, anomalies within telemetry can reflect sensor failure, transmission errors, and the major faults of the related subsystem. Therefore, anomaly detection for telemetry series has drawn great attention from the aerospace area, where probability prediction methods, e.g., Gaussian process regression and relevance vector machine, have an inherent advantage for anomaly detection in time series with uncertainty presentation. However, labelling a single point with probability prediction faces many isolated false alarms, as well as a lower detection rate for collective anomalies that significantly limits its practical application. Simple sliding window fusion can decrease the false positives, but the support number of anomalies within the sliding window is difficult to set effectively for different series. Therefore, in this work, fused with the probability prediction-based method, the Markov chain is designed to compute the support probability of each testing series to realize the improvement on collective anomaly mode. The experiments on simulated data sets and the actual telemetry series validated the effectiveness and applicability of our proposed method.

## 1. Introduction

Telemetry series, generally acquired by sensors and transmitted by telemetry links, are the only basis for the ground management system to judge the working performance and health status of orbiting spacecraft. The anomalies within the telemetry series generally reflect the transmission errors, sensor failure, and especially the critical faults of the related components [1,2]. For example, the battery performance degradation in electrical power subsystems (EPS) will cause an abnormal decrease of battery current; the power output of battery decreases, corresponding to the fault of a deplorable structure [3,4,5]. Therefore, anomaly detection for telemetry series has become a key step to identifying some potential failures to extend the life of the spacecraft. This work has also received great attention from many related research institutions, such as NASA, the European Space Agency, The University of Tokyo, and the United States Department of Defense [6,7,8]. Especially NASA has designed some tools, e.g., ORCA and the inductive monitoring system (IMS), to mine the anomalies within the telemetry series [9,10]. 

However, with the advantage of easy-to-perform and low computational complexity, the out-of-limitation (OOL) method remains popular for the ground monitoring of orbiting spacecraft [11]. The OOL method identifies abnormal points by comparing the real values and the preset thresholds. Obviously, many thresholds for different series need to be set in advance. With the rapid increase in the number of spacecraft and their telemetry series, manual workload increases. Moreover, OOL cannot detect the latent anomalies within the fixed thresholds that should be improved to meet the requirement of high reliability.

Thus, many data-driven methods with a strong learning ability have been proposed for anomaly detection in telemetry series. They can be roughly divided into three categories: The statistics-based method, distance-based method, and prediction-based method. The statistics-based method labels the points that do not obey normal data distribution or beyond the range of the statistical parameters [12]. Moreover, some statistical features can be extracted to describe the normal cases [13,14,15]. This type of method can only identify the statistical outliers without taking the time relation into the model. The distance-based method flags these points far from the normal data points or the normal clusters [16,17]. Nevertheless, this method is sensitive to the distance measure function, and it cannot detect the anomalies caused by the temporal context. The prediction-based method models the normal data with regression models, and outputs the predicted value for an unknown testing target. If the predicted error for a testing input is larger than that of the normal data, it will be regarded as an anomaly [18]. This method can model telemetry series; moreover, it has strong interpretability and can identify online anomalies. Especially with the rapid development of prediction methods, many of them, e.g., the least squares-support vector machine (LS-SVM) [19,20], relevance vector machine (RVM) [21], Gaussian process regression (GPR) [22], dynamic Bayesian network [23], and long short-term memory network [11], have been applied to realize anomaly detection. 

Furthermore, compared with some point prediction models, the probability prediction models, i.e., GPR and RVM, have an inherent advantage for anomaly detection. In detail, with the testing inputs, they can provide the mean and variance values under the Bayesian framework [24,25,26]. Then we can achieve the prediction interval (PI) with any coverage probability (CP) that can be set as the dynamic threshold for the testing targets. Therefore, the probability models referring to GPR and RVM are the focus of our work. Actually, not all of the factors in the real series can be modeled by the prediction model, so the labelling strategy of comparing a real value and the predicted output may face the challenge of some isolated false positives. Although these isolated false alarms do not happen frequently, they are widely distributed, which inevitably causes some extra work for the ground staff to eliminate the false alarms with expert experience. In particular, these will bring a lot of extra work in terms of ground monitoring, with an increasing number of telemetry series. Moreover, anomalies in real telemetry also happen collectively; the labelling strategy for a single point will cause missing alarms within the collective anomalies.

Therefore, in this work, a fusion method with a Markov chain model and probability prediction method is proposed for detecting the collective anomalies, with which the detection rate for the collective points is improved with the support probability computation. In addition, the false rates for the isolated points are mitigated with sliding window labelling. 

## 2. Related Works

Recently, some strategies have been designed to mitigate isolated false positives as well as improve the detection rate for collective anomalies. These strategies use sliding windows as the basic labelling unit, the prediction error and abnormal density of which are respectively computed to make judgments. For example, the maximum value of mean prediction errors for each training sliding window is used as the threshold to label the testing sliding window [27]. In addition, the percentage of decrease of the max prediction error at each step is computed for anomaly detection [11]. Furthermore, the support number of anomalous points within the sliding window can be set to control the labelling process [19]. The above strategies can mitigate false positives to some extent, while the density of anomalies is difficult to set effectively, and the statistical features of prediction errors are sensitive to some serious outliers. Therefore, in this work, fused with the labelling result generated by probability prediction models, i.e., GPR and RVM, we computed the support probability of each testing sliding window through a Markov chain model to mitigate isolated false positives, as well as improve the detection rate for collective anomalies.

Markov chain is a model of some random process that happens over time. Markov chains follow a rule called the Markov property. In particular, it is effective in detecting anomalies in cloud server systems as well as other anomaly detection areas, with the advantage of modeling each discrete transmission mode [28,29]. Therefore, in this work, the original time series was firstly processed to a discrete label series based on the detection result of the probability prediction-based method. Then, the Markov chain was modeled for computing the support probabilities of each testing sliding window. The testing series with the probability lower than the minimum probability of normal data sets will be anomalous. The experiments on the simulated data sets, i.e., Keogh data and Ma data, verified the effectiveness of the proposed method. In particular, the normal telemetry series validated its ability of mitigating the isolated false positives. More importantly, the case study on the actual telemetry series with anomalies showed its comprehensive performance of false rates and missing rates in the actual application.

## 3. Anomaly Detection with Probability Prediction Models

Compared with point prediction models that only output a single prediction value, probability prediction models can provide both the mean and variance value for each testing target. These outputs can easily construct the dynamic threshold that makes the probability model more suitable for anomaly detection. The typical and effective probability models refer to GPR and RVM. Both of them make a prediction based on statistical learning theory and the Bayesian inference framework. In this work, these two models were used to construct PIs to make judgments.

### 3.1. Probability Prediction with the Gaussian Process Regression Model

For the regression problem, the target variable is *y*, ***x*** is the *d* dimensional input variables, and the function relation is *f*(***x***), so:

(1) y=f(x)+ε,

where ε is the additive white noise and $\varepsilon ∼N(0,\sigma_{}^{2})$.

For each input $x_{i}$, $f(x_{i})$ is a random variable. The Gaussian process model makes one assumption that these function values obey a multivariate normal distribution. Namely, $f(x_{1}),\ldots ,f(x_{N})$ with different input samples obey to joint Gaussian distribution [24]. Then, the function distribution forms a Gaussian process:

(2) $$f(x)∼GP(m(x_{i}),k(x_{i},x_{j})),$$

where $m(x_{i})$ is the mean function and $k(x_{i},x_{j})$ is the covariance function. They are derived by Equations (3) and (4):

(3) $$m(x_{i})=E[f(x_{i})],$$

(4) $$k(x_{i},x_{j})=E[(f(x_{i})-m(x_{j})(f(x_{i})-m(x_{j}))],$$

where $x_{i}$ and $x_{j}$ are different input samples. *E*() is the expectation function. $k(x_{i},x_{j})$ describes the relation between the input samples $x_{i}$ and $x_{j}$. A typical covariance function is the square exponential function defined by Equation (5) [25]:

(5) $$k(x_{i},x_{j})=\sigma_{s}{}^{2}\exp\left\{-\sum_{l=1}^{d}\frac{{\left(x_{i}-x_{j}\right)}^{2}}{2\omega_{l}}\right\},$$

where $\sigma_{s}{}^{2}$ is a width parameter which indicates the uncertainty of unknown function and $\omega_{l}$ is the length parameter controlling the delay speed of the exponential function. When $x_{i}$ is similar to $x_{j}$, the exponential function value is close to 1. This covariance function makes the closer points have a higher relation.

Another assumption about the GPR model is that the target value *y* is independent of the function *f(**x**)* and the noise distribution is independent with each other. Thus, with Equation (1), *y* also obeys a Gaussian process:

(6) $$y∼GP(m(x_{i}),k(x_{i},x_{j})\;+\sigma_{n}^{2}\delta_{ij}),$$

where $\sigma_{n}^{2}$ is the noise variance of ε. $\delta_{ij}$ is the Dirac function, $\delta_{ij}=1$ only when i=j.

With the Gaussian process property that the target values ***y*** with the training input and the function value $f_{*}$ with the testing input also obey a GP:

(7) $$\left(\begin{array}{l} y\\ f_{*} \end{array}\right)∼\left(\left[\begin{array}{c} m(x)\\ m(x_{*}) \end{array}\right],\left(\begin{array}{cc} C(x,x) & K(x,x_{*})\\ K{\left(x,x_{*}\right)}^{T} & K(x_{*},x_{*}) \end{array}\right)\right),$$

where m(x) is the mean vector for the training samples and $m(x_{*})$ is the mean vector for the testing inputs. If there is only one testing input, $m(x_{*})$ is a value. C(x,x) is the covariance matrix of the training data itself and includes the noise variance interference, $C(x,x)=K(x,x)+\sigma_{n}^{2}$. $K(x,x_{*})$ is the covariance matrix of the training data and the testing input. $K(x_{*},x_{*})$ is the covariance of the testing itself.

Based on Equation (7) and the property of GP, $f(x_{1}),f(x_{2}),f(x_{3}),\ldots ,f(x_{N}),f(x_{*})$ form a multivariate Gaussian distribution. When $f(x_{1}),f(x_{2}),f(x_{3}),\ldots ,f(x_{N})$ is known (in Equation (7), the target value is known, and it is derived by the corresponding function value with the added white noise), the property of $f(x_{*})$ can be derived by the mean function and the variance function. Namely:

(8) $$\bar{f_{*}}=m(x_{*})+K(x,x_{*})C{\left(x,x\right)}^{-1}(y-m(x)),$$

(9) $$\mathrm{cov}(f_{*})=K(x_{*},x_{*})-K(x,x_{*})C{\left(x,x\right)}^{-1}K{\left(x,x_{*}\right)}^{T},$$

where $\bar{f_{*}}$ is the mean value for the testing target and $\mathrm{cov}(f_{*})$ is the variance of the function value. The PI for a testing target is $PI_{f_{*}}=[\bar{f}(x_{*})-\beta \times \sqrt{\mathrm{cov}(f_{*})+\delta_{n}{}^{2}},\bar{f}(x_{*})+\beta \times \sqrt{\mathrm{cov}(f_{*})+\delta_{n}{}^{2}}]$. $\delta_{n}$ is the standard variance of the additive noise, β is the uppermost quantile of the normal distribution with the given CP. Noted that the traditional confidence interval of a prediction model just provides bounds for the population mean [30]. As a comparison, the PI is an estimate of an interval that one observation sample will fall into with a certain probability. Namely, the PI with the injected noise variance is much wider than the confidence interval with the same CP. Evidently, for the application of anomaly detection that needs to make a judgment for each observation, the traditional confidence interval is less effective than the PI that contains the noise interference as shown in the added $\delta_{n}{}^{2}$ in the PI equation of GPR.

In Equations (8) and (9), the unknown parameters, called hyperparameters, can be optimized under the Bayesian framework through a maximum-likelihood function estimation. In the real prediction, the normalization preprocess can be generally performed on the training data set. Thus, the mean function can be set to zero function. In addition, the hyperparameters within the covariance function and likelihood function can be optimized with the conjugate gradient descent method [25]. 

### 3.2. Probability Prediction with the Relevance Vector Machine Model

For the regression problem described by Equation (1), with the testing input $x_{*}$, RVM has the same function type with the SVM model shown in Equation (10):

(10) $$f(x_{*})=\sum_{i=1}^{N}\omega_{i}K(x_{*},x_{i})+\omega_{0},$$

where $K(x_{*},x_{i})$ is the kernel function which has the same meaning with that of GPR to measure the relation between the input samples. $x_{i}$ is the *i*th training input. $\omega_{i}$ is the weight for the kernel of the *i*th training data. In addition, the size of the training sample is *N*, and the dimension of each testing sample is *d*. $\omega_{0}$ is a constant term.

With the independent assumption of *y* and *f*(***x***), $p(y|x)=N(f(x),\sigma_{n}{}^{2})$, the likelihood of the training data can be derived:

(11) $$\begin{array}{cc} p(y|\omega ,\sigma_{n}^{2}) & ={\left(2\pi \sigma_{n}^{2}\right)}^{-N/2}\exp\{-{\left\|y-f(x)\right\|}^{2}/(2\sigma_{n}^{2})\}\\ & ={\left(2\pi \sigma_{n}^{2}\right)}^{-N/2}\exp\{-{\left\|y-\Phi \omega \right\|}^{2}/(2\sigma_{n}^{2})\} \end{array}$$

where $y={\left(y_{1},\cdot \cdot \cdot ,y_{N}\right)}^{T}$, $\omega ={\left(\omega_{0},\cdot \cdot \cdot ,\omega_{N}\right)}^{T}$, and Φ is the kernel function matrix, $\Phi ={\left[\phi (x_{1}),\phi (x_{2})\cdot \cdot \cdot \phi (x_{N})\right]}^{T}$, $\phi (x_{i})=\left[1,K(x_{i},x_{1}),\cdot \cdot \cdot ,K(x_{i},x_{N})\right]$, and the size of Φ is N×(N+1).

The unknown parameters in Equation (11) are the weights that can be directly optimized through maximum-likelihood estimation. However, this operation may cause a serious overfit problem. In detail, there are *N* training samples, and the size of the unknown weight is *N* + 1. Therefore, in order to make limitations on these weights, Tipping defined a zero-mean Gaussian prior distribution, $N(0,\alpha_{i}^{-1})$ over $\omega_{i}$ [26]; thus:

(12) $$p(\omega |\alpha )=\prod_{i=0}^{N}N(\omega_{i}|0,\alpha_{i}^{-1})=\prod_{i=0}^{N}\frac{\alpha_{i}}{\sqrt{2\pi }}\exp(\frac{\omega_{i}^{2}\alpha_{i}}{2}),$$

where α is the hyperparameter vector within the Gaussian prior distribution, $\alpha =\{\alpha_{0},\alpha_{1},\cdot \cdot \cdot ,\alpha_{N}\}$. Obviously, the hyperparameters α have a one-to-one mapping relation with the weight vector ω. In particular, by controlling the influence on the weights with the hyperparameters in Gaussian prior distribution, the sparsity of the model can be realized, which is the main advantage of the RVM model.

Suppose the hyperparameters and the noise variance $\sigma_{n}^{2}$ obey the Gamma prior distributions:

(13) $$\begin{array}{l} p(\alpha )=\prod_{i=0}^{N}\mathrm{Gamma}(\alpha_{i}|a,b)\\ p(\sigma_{n}^{2})=\prod_{i=0}^{N}\mathrm{Gamma}(\beta |c,d) \end{array}$$

where $\mathrm{Gamma}(\alpha_{i}|a,b)=\Gamma {\left(\alpha \right)}^{-1}b^{a}\alpha^{a-1}e^{-b\alpha }$ and $\Gamma (\alpha )=\int_{0}^{\infty }t^{a-1}e^{-t}dt$. *a* and *c* is the shape parameter of Gamma distribution, while *b* and *d* is the scale parameter of Gamma distribution. 

Based on Bayesian theory, Equation (14) can be derived:

(14) $$p(\omega ,\alpha ,\sigma_{n}^{2}|y)=\frac{p(y|\omega ,\alpha ,\sigma_{n}^{2})\cdot p(\omega ,\alpha ,\sigma_{n}^{2})}{p(y)},$$

where the marginal distribution $p(y)=\int p(y|\omega ,\alpha ,\sigma_{n}^{2})\cdot p(\omega ,\alpha ,\sigma_{n}^{2})d\omega d\alpha d\sigma^{2}$.

Therefore, the likelihood distribution of hyperparameters is obtained as Equation (15):

(15) $$\begin{array}{l} p(y|\alpha ,\sigma_{n}^{2})=N(0,C)\\ ={\left(2\pi \right)}^{-N/2}{\left|\sigma^{2}I+\Phi A^{-1}\Phi^{T}\right|}^{-1/2}\exp\left\{-\frac{1}{2}y^{T}{\left(\sigma^{2}I+\Phi A^{-1}\Phi^{T}\right)}^{-1}y\right\} \end{array}$$

where $A=diag(\alpha_{0},\alpha_{1},\cdot \cdot \cdot ,\alpha_{N})$, and the hyperparameters α and $\sigma^{2}$ are estimated by iteration, which is not described detailed in this section. Please refer to Reference [26] to find the detailed computing process.

For a testing input $x_{*}$, the mean and the variance are derived by the Equations (16) and (17):

(16) $$\mu_{*}=\mu^{T}\phi (x_{*}),$$

(17) $$\sigma_{*}^{2}=\sigma_{MP}^{2}+\phi {\left(x_{*}\right)}^{T}\sum \phi (x_{*}).$$

The noise includes two parts; $\sigma_{MP}^{2}$ is the estimated noise variance derived by the model training. $\phi {\left(x_{*}\right)}^{T}\sum \phi (x_{*})$ reflects the uncertainty of weights estimation. Finally, PI of RVM can be constructed as $\left[\mu_{*}-\beta \times \sqrt{\sigma_{*}^{2}},\mu_{*}+\beta \times \sqrt{\sigma_{*}^{2}}\right]$.

### 3.3. Anomaly Detection with Prediction Interval Constructed by Probability Prediction Model

**Figure 1:**
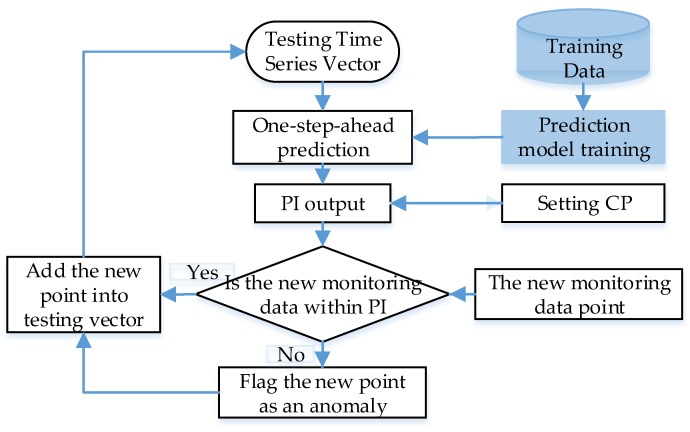
Anomaly detection with the probability prediction model.

Based on the introduction of Section 3.1 and Section 3.2, the GPR and RVM model can output the mean and the variance value simultaneously. Then, the PI can be constructed with them under a certain CP. The detection flowchart based on the probability prediction model is given in Figure 1.

As shown in Figure 1, the anomaly detection process includes two parts, i.e., the phases of training and testing. 

At the training phase, the main operation procedures refer to data processing, input data construction, and prediction model training. For data processing, normalization and error data deletion can be performed on the original samples. In addition, autocorrelation analysis is applied to realize the input data construction. Based on the available training data samples, the hyperparameters and noise variance in the GPR and RVM are optimized under the Bayesian framework. 

At the testing phase, with the testing input, the trained one-step-ahead probability prediction model can output the predicted mean and variance. Then, the PI constructed with the setting CP at each step will be set as the dynamic threshold to label each testing target. If the new monitoring data point beyond this PI, it will be regarded as an anomaly. The testing process can perform online with the continuous testing input.

### 3.4. Problem Formulation

**Figure 2:**
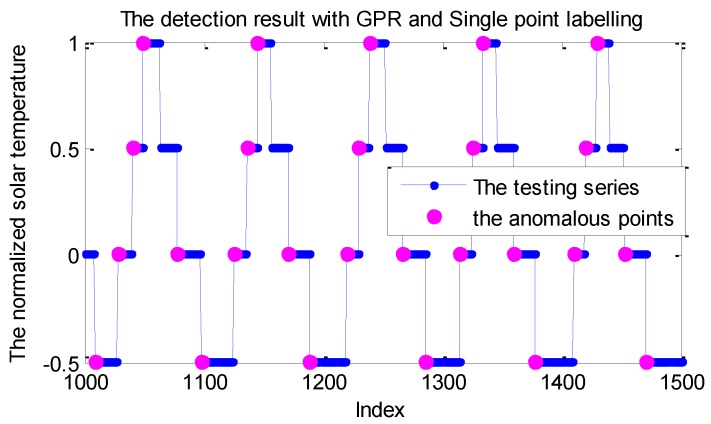
One labelling example for the solar temperature series with the Gaussian process regression (GPR) model.

With the detection framework given in Figure 1, we can flag each point continuously; one labelling example is shown in Figure 2.

As shown in Figure 2, the series is the real solar temperature telemetry from the EPS of a spacecraft that is labeled by the GPR model, where some isolated points are labeled as anomalies. Nevertheless, in the real application, the significant abnormal patterns always show the persistence property over a period. Namely, the isolated points beyond the PI within the telemetry series are normal from the view of fault analysis. These false alarms are mainly caused by the inaccurate modeling for the irregular mode switch. Although these false positives are relatively smaller compared with the large scale of the testing points, the ground operation staff have to check the telemetry status to exclude these false alarms. Obviously, these false positives bring a lot of extra work that largely affects the applicability of the monitoring method.

In this case, we can set the support number of the abnormal points within the sliding window to mitigate the isolated false alarms. However, the number, set by users, cannot be effectively determined, which has a serious impact on the detection results. Moreover, this labelling strategy is unable to model the label distribution of the points within the testing sliding window. 

Thus, in this work, the Markov chain model was designed to fuse with the probability prediction model to realize the anomaly detection of the telemetry series. Firstly, the probability prediction model makes the original continuous samples change into the discrete values. Then, the Markov chain model is applied to model the state transmission probability, where the transmission probability of normal mode is estimated with the normal validation data. Consequently, the abnormal mode can be labeled by the Markov chain. Moreover, there is no need to set the number of abnormal data within the sliding window at the testing phase, which enhances the robustness of the detection model. The detection method with probability prediction and the Markov chain is described in Section 4 in detail.

## 4. Markov Chain Labelling Fused with Probability Prediction-Based Method

### 4.1. Markov Chain Model

The Markov process is a type of random process, where there is a transition probability that the system transmits from one state to the other state. Thus, the Markov model can be represented by three tuples {S,P,Q}. S is the state space that has a finite number of states, represented by $S=\{s_{1},s_{2},s_{3},\ldots ,s_{m}\}$. P is the transmission matrix between different states. Q is the initial probability of the related states. For the Markov model, there are two important assumptions: The Markov property and time-homogeneous assumption [28]. 

The Markov chain model is the discrete-time and discrete-state Markov process [29]. For the finite states, their initial probability vector is Q. The corresponding relation is $P(s_{i})=q_{i},i=1,2,3,\ldots ,m$, and the transmission matrix $P={\left[P_{ij}\right]}_{m\times m}$, where $P_{ij}=P(x_{n}=s_{j}|X_{n-1}=s_{i})$. For a new testing sequence $Y=\{y_{1},y_{2},\ldots ,y_{N1}\}$, the support probability for it can be computed by the product of the initial probability and the success transmission probability as shown in Equation (18):

(18) $$P(y_{1},y_{2},\ldots ,y_{N_{Y}})=\{\begin{array}{cc} q_{y_{1}} & N_{Y}=1\\ q_{y_{1}}\prod_{n=2}^{N_{Y}}p_{y_{n-1}y_{n}} & N_{Y}\geq 2 \end{array}.$$

As described above, it is the first-order Markov chain. For the high-order Markov chain model, the computing equations for the initial probability and transmission matrix are similar.

### 4.2. Markov Chain Training for Normal Series Labeled by the Probability Prediction Model

For a point of a time series at time *t*, denoted as *x*_t_, the PI for it is [L_i_, U_i_], which is derived by a probability prediction model. If *x*_t_ lies out of the PI, its label is 1; otherwise, the label is 0. Therefore, for the label state space, there are only two states referring to 0 and 1. With the labelling process based on the probability prediction model, the testing subsequence can be changed to the label series. With the segmentation of the sliding window for the label series, it will produce many subseries containing only 0 and 1.

Then, a Markov chain model can be performed on this label series, where the state space has only two states, 0 and 1. For a first-order Markov chain, the initial probability can be computed by Equation (19):

(19) $$q_{i}=N_{i}/N,\;i=0,1,$$

where *N* is the size of the testing series. $N_{i}$ is the number of state *i*. The sum of initial probability is 1. In addition, the size of the transmission matrix is 2, which can be computed by Equation (20).

(20) $$p_{=}\left[\begin{array}{cc} p_{00} & p_{01}\\ p_{10} & p_{11} \end{array}\right]$$

The elements in the transmission matrix are defined by Equation (21):

(21) $$p_{ij}=N_{ij}/(N-1),\;i=\{0,1\},\;j=\{0,1\}.$$

Thus, for a testing subseries Y with the size of *Ny*, the support probability can be computed by Equation (21). 

**Figure 3:**
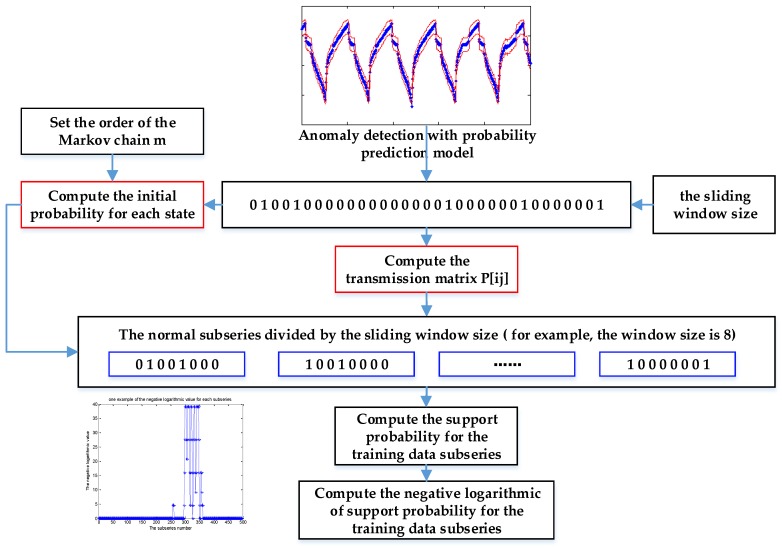
Markov chain training for normal available data.

Then, we can realize the Markov chain modeling with the normal available data series, as shown in Figure 3.

As shown in Figure 3, the original normal series are firstly processed to a label series based on the predicted results. The initial probability of normal and anomaly, as well as the transmission matrix, are derived with the label series. Then, the label series is segmented by a sliding window, where the sliding window size is set by users. In particular, the sliding window size should be larger than the number of continuous abnormal points among the training data to make the labelling strategy effective for the testing series. The support probability for each label subseries is computed with the initial probability and the transmission matrix. Furthermore, the negative logarithmic value is applied to replace the original support probability, which may be relatively small to make a comparison and figure out. Based on the training of Markov chain with the available normal data, the maximum negative logarithmic value of support probability is set as the threshold for making judgments on the testing input. There are two reasons to make this setting effective. Firstly, the maximum negative logarithmic value of support probability corresponds to the lowest support probability of a sliding window subseries. Secondly, there is a prerequisite that the validation data be adequate and normal without the interference of error points and abnormal mode. Thus, the maximum negative logarithmic value of support probability reflects the lowest probability of sliding window subseries under a normal condition. As a result, if the support probability of a testing sliding window is lower than the lowest probability of normal sliding data, namely, it has a higher negative logarithmic value of support probability than this setting threshold, it may be an abnormal mode with a higher probability. 

### 4.3. Anomaly Detection with Markov Chain Fused with Probability Prediction-Based Method

**Figure 4:**
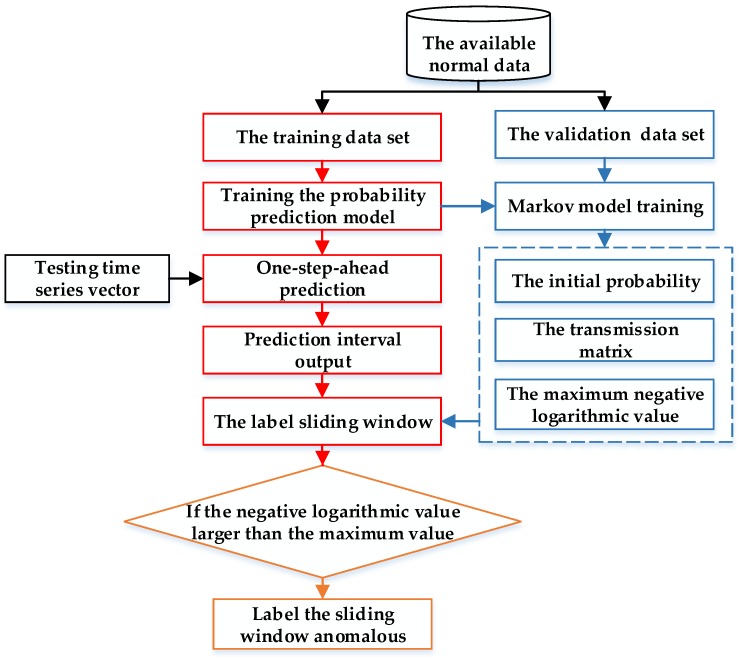
The proposed anomaly detection with the fusion of probability prediction and Markov chain model.

As described in Section 3, based on the available normal data, the one-step-ahead probability prediction model can be derived based on GPR or RVM. Then, the normal validation data series can be transformed to the label series based on the prediction results. However, with the exiting unpredictable factors, some false alarms happen on some isolated samples with the independent labelling strategy for each point. Hence, in this work, the Markov chain was realized to decrease the false positives on isolated points as well as improve the detection rate for collective anomalies. The detection method is shown in Figure 4.

As shown in Figure 4, this method includes three parts: The training for the probability prediction model, the Markov chain model training, and the testing phase.

1. Probability model training

In this work, the probability prediction models refer to GPR and RVM model. In addition, the available normal data set is divided into two parts to train the prediction model and the Markov chain model, respectively. 

2. Markov chain model training

For the Markov chain training, the labels for each validation sample form a label series. The outputs of Markov chain training contain the initial probability for each state, the transmission matrix, and the max negative logarithmic value. 

3. Testing phase

For a testing time vector, it is firstly used as the input of the one-step-prediction model to obtain its PI. Then, the label value is added into the sliding window of label subseries. With the initial probability and the transmission matrix, the support probability for the sliding window subseries can be derived. If the support probability is larger than the maximum value of the training data, the related testing series window will be flagged anomalous.

## 5. Experimental Results and Analysis

Given that the key telemetry series, acquired by sensors of orbiting spacecraft, are generally the pseudoperiod sequences with the influence of regular orbit and working mode, in order to evaluate the performance of the proposed method quantitatively, the commonly used simulated data sets, i.e., the Keogh data and Ma data, which have similar properties with the telemetry series, are first applied in this section. In particular, the labelling strategies referring to single point labelling strategy and sliding window fusion strategy were realized to make comparisons, where single point labelling represents the original detection with the probability prediction model. Additionally, the sliding window fusion refers to the detection strategy labeled by anomaly density [19,31], which makes judgments based on the support number of the abnormal points within the sliding window. If the abnormal number of the points within the sliding window is larger than the setting support number, this sliding window will be labeled anomalous. The estimation indices include the detection rate (DR) and the false positive rate (FPR).

Furthermore, the applicability of the proposed method for the anomaly detection of the telemetry series was validated from two aspects. The experiments on normal telemetry series from EPS estimated the performance of these methods on isolated false positives. Furthermore, the telemetry series with the real anomalies was used as a case study to test the anomaly detection ability of the proposed method in the actual application. 

### 5.1. Experiments on Simulated Data Sets

Keogh Data were designed to test the performance of three anomaly detection methods, including Immunology (IMM), a wavelet-based tree structure (TSA-Tree) and Tarzan, in Reference [32]. They have since been applied in many studies [33,34]. The normal series of Keogh Data, Y_1_, is generated by Equation (22):

(22) $$Y_{1}=\sin(\frac{50\pi }{N}t)+n(t)+e_{1}(t),$$

where the size of series, *N*, is set to 1200. n(t) is the addictive white Gaussian noise with zero mean and standard variance 0.1. $e_{1}(t)$ is the injected abnormal mode at the indices from 800 to 832 defined by Equation (23):

(23) $$e_{1}(t)=\{\begin{array}{c} \sin(\frac{75\pi }{N}t)-\sin(\frac{50\pi }{N}t),t\in [800,832]\\ 0,\;otherwise \end{array}.$$

**Figure 5:**
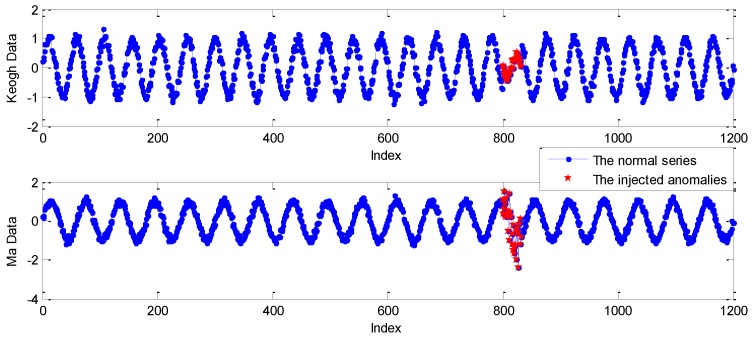
One example of Keogh Data and Ma Data.

In addition, Ma Data are also a simulated series designed to test the Support vector regression (SVR) algorithm [35]. It is defined by Equation (24):

(24) $$Y_{2}=\sin(\frac{40\pi }{N}t)+n(t)+e_{2}(t),$$

where *N* is also set to 1200. n(t) is the white noise with zero mean and variance 0.1. The abnormal mode of $e_{2}(t)$ is the added Gaussian white noise with zero mean and variance 0.5, injected at the same indices as that of Keogh Data. One example of Keogh Data and Ma Data is shown in Figure 5.

As shown in Figure 5, the blue curves marked with point are the normal series of Keogh Data and Ma Data, where the points labeled red star are the injected anomalies. Obviously, Keogh Data show an abnormal pattern in the length of period. However, the anomalies of Ma Data are mainly caused by high variance noise.

In this part, three labelling strategies, i.e., single point labelling, sliding window fusion, and Markov chain, were performed on these two simulated data sets. The probability prediction models were GPR and RVM algorithms where the CP is 95%. The initial hyperparameters of the GPR model were set to some random values between 0 and 1. The kernel width of RVM model was set to 8. For different application areas, the sliding window size was set according to the detection requirement. For example, the minimum attack time was set as the sliding window size to detect the network attacks [31]. The size can also be set combined with the sample rate and time interval [11]. However, it is hard to determine an effective length for different abnormal modes from a theoretical view. Additionally, the length of abnormal mode cannot be determined in advance. Thus, in this part, as the injected abnormal length is 32, the sliding window size ranges from 5 to 10 were set to cover a part of the abnormal mode to provide a comprehensive estimation with the detection indices of DR and FPR.

The upper limitation of the abnormal number for the sliding window fusion strategy was half of the window size plus 1. If the window size is an odd number, the number of the abnormal point is set to be the larger integer, smaller than half of the window size plus 1. For example, if the window size is 7, the support number of abnormal points is 4. If the window size is 8, this will be 5. Obviously, the detection result of sliding window fusion is very sensitive to the support number. The first-order Markov chain model is the focus in these experiments.

**(1) The Detection Results for the Series of Keogh Data with the GPR Model**

**Figure 6:**
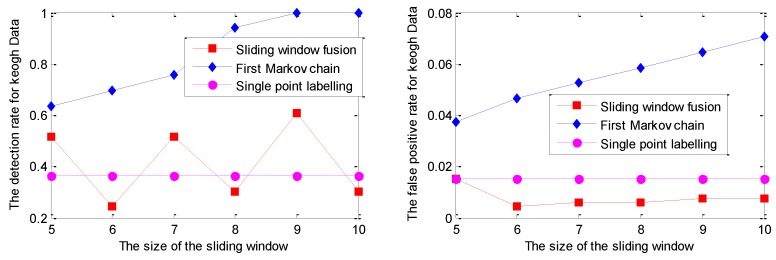
The detection results with the GPR model fused with different labelling strategies under different sizes of the sliding window.

The detection results for one series of Keogh Data with the GPR model under different sliding window lengths are given in Figure 6. It is noted that the series of Keogh Data has the nature of randomness; similar experiments have been done several times, but only one of them is given in this work due to space limit. Similar conclusions could be made based on the other experiments.

As shown in Figure 6, the DR of the Markov chain is better than that of the other two strategies. Moreover, with the increment of the sliding window size, the DR of the Markov chain has become 100% for the sliding window size of 9 and 10. Correspondingly, the FPR of the Markov chain increases with the incremental sliding window size, which is larger than that of the other two strategies. Evidently, both DR and FPR of single point labelling are not sensitive to the sliding window size. 

**Figure 7:**
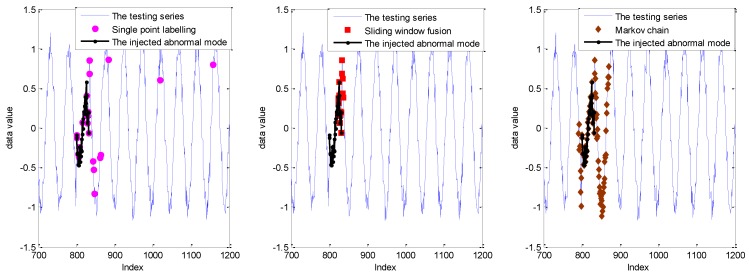
The detection results for the Keogh Data with three labelling strategies under the sliding window size of 10.

In order to make a better analysis, the detection results with the sliding window size of 10 are given in Figure 7.

As shown in Figure 7, the detection result with the single point labelling strategy has three isolated false alarms. Furthermore, some abnormal points in the collective abnormal mode are impossible to label. As a comparison, with the introduction of sliding window, these isolated points are mitigated with sliding window fusion and the Markov chain model. However, some points within collective anomalies cannot be detected with the sliding window fusion. In other words, the number of abnormal points within the related sliding window is smaller than the setting support number of 6. Evidently, the complete abnormal points from the indices of 800 to 832 are labeled accurately by the fusion with the Markov chain and probability prediction model. Furthermore, the false alarms caused by the Markov chain are concentrated around the abnormal mode with the inference of the sliding window size. 

**(2) The Detection Results for the Series of Keogh Data with the RVM Model**

**Figure 8:**
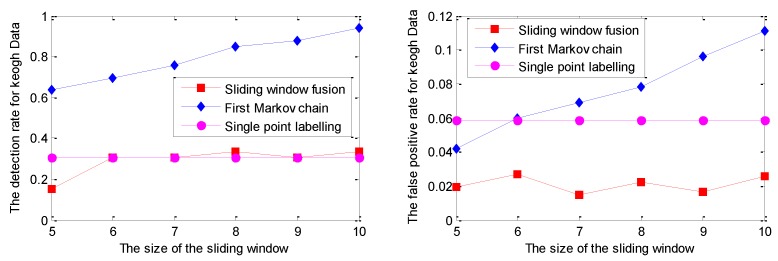
The detection results for one series of the Keogh Data with the relevance vector machine (RVM) model.

With similar parameter settings to those described at the beginning of this subsection, the experimental results of DR and FPR on the Keogh Data with the RVM model under different window sizes are given in Figure 8.

**Figure 9:**
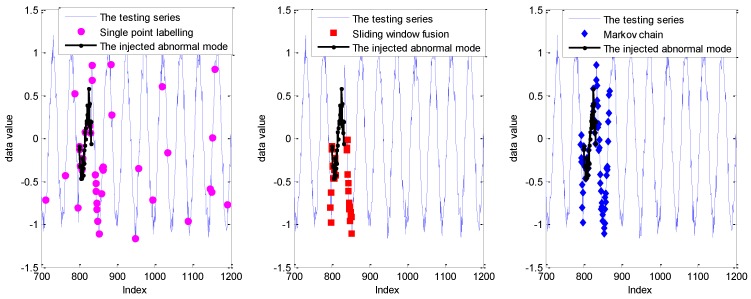
The detection results for the Keogh Data based on the RVM model with three labelling strategies under the sliding window size of 10.

As shown in Figure 8, the detection performance of sliding window fusion and the Markov chain is sensitive to the size of the sliding window size. Moreover, the detection with sliding window fusion shows a lower DR, which is largely affected by the setting support number. However, it is hard to set it appropriately in the real application for different abnormal modes. The detection results with a sliding window size of 10 are given in Figure 9.

As shown in Figure 9, compared with the GPR prediction model, more isolated points are labeled anomalous, which mainly depends on the PI performance. Similarly, the abnormal points within the collective mode at the indices from 800 to 832 cannot be detected completely with the strategy of single point labelling and sliding window fusion. The detection with sliding window fusion can mitigate the isolated false alarms with the introduction of the sliding window. Thus, the FPR of sliding window fusion in Figure 8 is smaller than that labeled by single point. Nevertheless, sliding window fusion cannot model the transmission relation of the points within the sliding window. Thus, the DR is worse than that with the Markov chain. By contrast, detection with the Markov chain mitigates the isolated false alarms and improves the DR for the collective anomalies. Nevertheless, some points around the collective anomalies are also be regarded as anomalies with the influence of the sliding window.

**(3) The Detection Results for the Series of Ma Data with the GPR Model**

**Figure 10:**
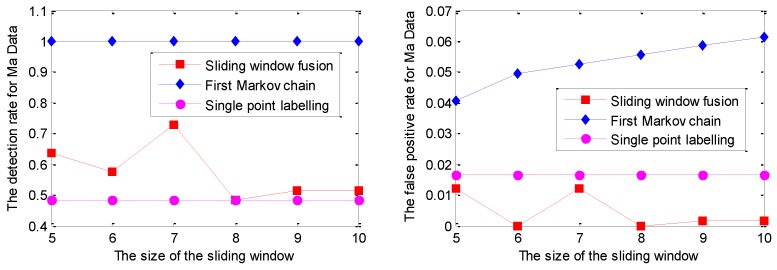
The detection results for Ma Data with the GPR model under different sliding window sizes.

The similar detection results for the Ma Data series based on GPR are given in Figure 10.

**Figure 11:**
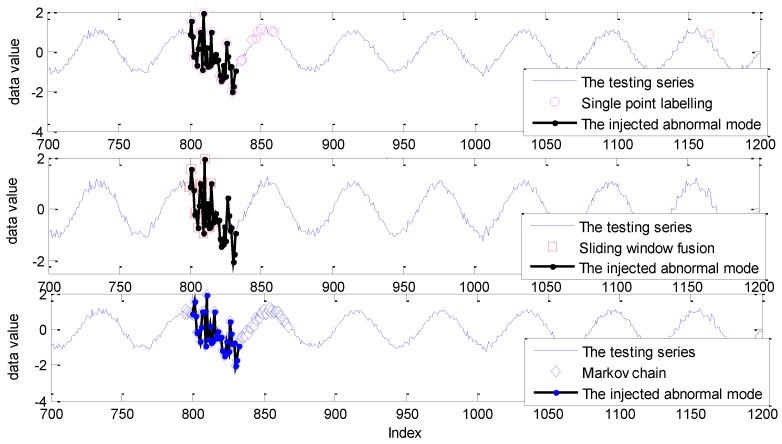
The detection results for the Ma Data based on the GPR model with three labelling strategies under the sliding window size of 10.

As shown in Figure 10, for the sliding window size ranging from 5 to 10, the DR of single point labelling is close to 0.5, while the DR of the Markov chain is up to 100%. In other words, the missing alarms, up to half of the abnormal mode, caused by single point labelling, are successfully identified by the realization of the Markov chain. In addition, the DR of sliding window fusion fluctuates from 51% to 72%. The FPR of the Markov chain model is also larger than the other two strategies with the influence of labelling each sliding window, not a single point. The detection results are given in Figure 11.

As shown in Figure 11, a similar conclusion can be derived: The FPR of single point labelling is generally from the isolated normal indices far from the abnormal indices, while the false alarms of sliding window fusion and the Markov chain aggregate in the indexes around the abnormal mode. 

**(4) The Detection Results for the Series of Ma Data with the RVM Model**

**Figure 12:**
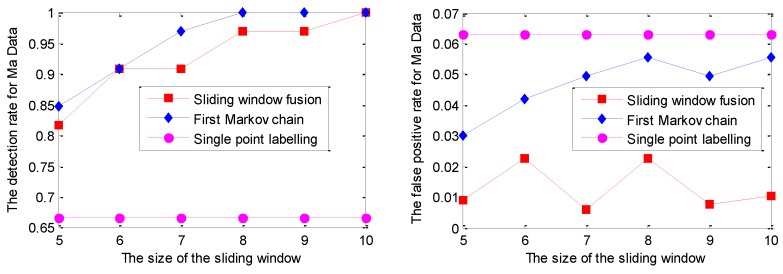
The detection results for Ma Data with the GPR model under different sliding window sizes.

Similarly, the RVM model is applied to perform prediction on Ma Data; the detection curves of DR and FPR for Ma Data with the RVM model are given in Figure 12.

**Figure 13:**
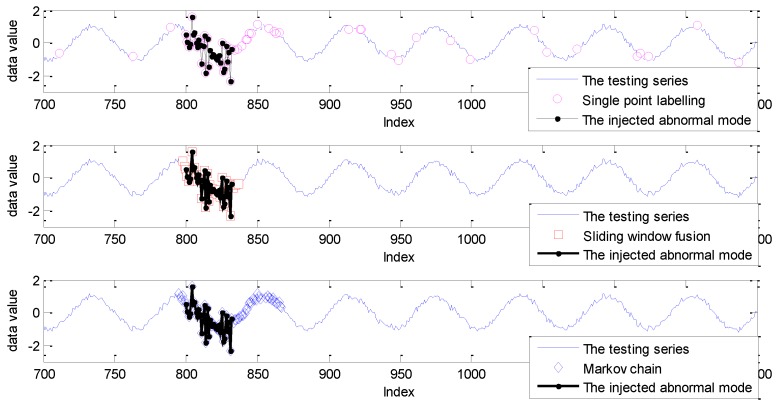
The detection results for the Ma Data based on the RVM model with three labelling strategies under the sliding window size of 10.

As shown in Figure 12, the DR of sliding window fusion and the Markov chain are similar under different sliding window sizes. The main reason is that the DR of single point labelling has reached 65%. In other words, more than half of the points in the collective mode can be effectively labeled with the RVM prediction model that makes the DR of sliding window fusion increase under different sliding window sizes. Note that the FPR of single point labelling is larger than that of the other two strategies. The detailed detection results based on the RVM model for Ma Data are shown in Figure 13.

As shown in Figure 13, some isolated points marked with the RVM prediction model can be mitigated with the strategies of sliding window fusion and the Markov chain. In particular, the strategy of sliding window fusion can label the collective mode successfully with a lower FPR under the sliding window size of 10. It is noted that with different sliding window sizes, the DR of the Markov chain is generally better than that of sliding window fusion. These detailed figures are not given in this work. Moreover, some points around the abnormal mode are labeled anomalous, which causes the FPR increase of Markov chain. In reality, with the entering of the abnormal samples into the testing input of the prediction model, it is inevitable to label some normal points close to the abnormal mode with the prediction-based anomaly detection.

For the experiments on the Keogh Data and Ma Data with the GPR and RVM model, the quantitative results about DR and FPR are given in Table 1.

As shown in Table 1, for the lower DR of the prediction model for the injected mode, the strength of the Markov chain model is obvious, which can model the mode transmission of the points in the testing sliding window. For example, the DR of single point labelling with the GPR model for Keogh Data is only 36.36%, while the DR of the Markov chain model has reached 100%. Similar cases are also shown on the Keogh Data with the RVM model as well as on the Ma Data with the GPR model. Nevertheless, with the increase of DR with single point labelling, the advantage of the Markov chain is weakened, as shown in the detection result for Ma Data with the RVM model. 

However, as the detection result with the Markov chain is sensitive to the sliding window size, the indicator of true skill statistic (TSS) defined by the difference between DR and FPR is computed to provide a reference on the choice of sliding window [36]. The detection with a larger TSS value shows a better performance. The TSS results are given in Table 2. 

As shown in Table 2, for the Keogh Data with the GPR model, with a sliding window size from 5 to 10, the optimal sliding window size with the best TSS value is 8, while it is 10 for the Keogh Data with the RVM model. In addition, for the Ma Data, the optimal window size is 5 and 9, respectively, with the GPR and RVM model. Obviously, it is hard to set an optimal sliding window size for different data series and prediction models. As the prediction interval of GPR is larger than that of the RVM model with the same CP, the sliding window subseries with relatively small abnormal points can be detected effectively with the GPR model. This makes the optimal sliding window size of GPR smaller than that of the RVM model. In other words, if the length of the abnormal mode is available, the sliding window size of the GPR model can be set one fourth of the abnormal length. As a comparison, it should be set one third of the length for the RVM model. Noted that the sliding window size can also be set to another value to make experiments. What is more, it should be determined according to the actual requirement. 

### 5.2. Experiments on Normal Telemetry Series

The telemetry series with pseudoperiodicity is the only basis for the ground monitoring system to judge the working performance and health status of a spacecraft. Among the complex systems, the EPS is a key system that generates, moderates, and provides energies for other systems [3]. EPS makes a big difference on the success of the mission. Generally, the effective telemetry series in the EPS are voltage, current, and temperature, reflecting the performance of the solar array, battery, charging controller, discharging controller, and the shunt-regulator. 

In this part, given that detection with the RVM model can reach a similar conclusion to that with the GPR model, GPR is applied as the main probability prediction model to control the length of this work. Detection with single point labelling may cause some false positives at the phase of mode change, such as the phase from the shadow period to the sunlight period. These false positives will bring some extra work to the ground staff. Therefore, in this part, the performance of false alarms for some isolated points is tested with different labelling strategies.

**Figure 14:**
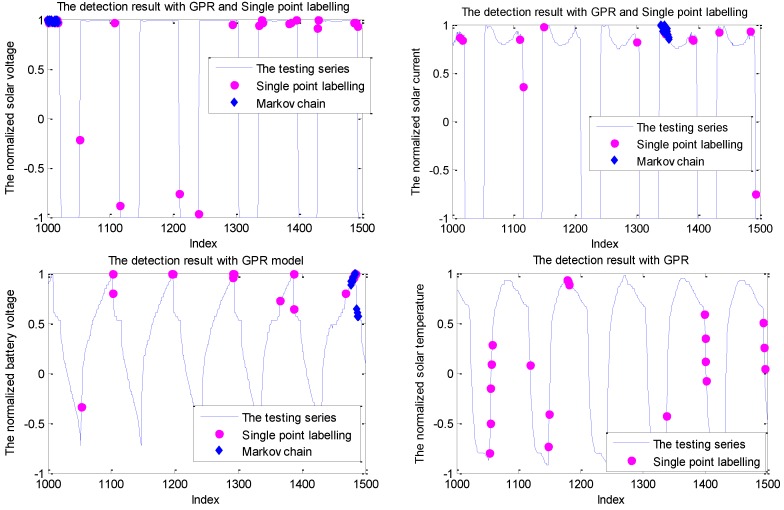
The detection results for the telemetry series with the GPR model.

The normal telemetry series exactly include the solar voltage, solar current, battery voltage, and battery temperature. The parameters of the three strategies remain the same with the simulated experiments. Additionally, the embedded dimension is determined by autocorrelation analysis. The detection results for these telemetries with the sliding window size of 10 are shown in Figure 14.

As shown in Figure 14, the solar current and solar voltage have two stable states referring to the shadow period and sunlight period. Between these two stable modes, there are transition stages from the shadow period to the sunlight or change from the sunlight to the shadow. Due to the influence of orbit and working mode, these telemetry series show the pseudoperiod property. However, with the influence of the space environment and collecting noise, there is some uncertainty regarding the value and period of these telemetry series. Evidently, this cannot be accurately modeled, which may cause some false alarms around the transition stage. These single false positives are mitigated by the Markov chain and sliding window fusion. Similar to the experiments on the simulated data, some points of solar voltage around the index 1000 are labeled anomalous with the Markov chain because of its strong detection ability for mode change. A similar conclusion can be derived through other telemetry series.

It is noted that there are no false alarms with the detection of sliding window fusion under a sliding window size of 10. In other words, none of the testing sliding windows has more than 6 abnormal points.

In these experiments, we also set the sliding window size from 5 to 10. The quantitative detection results are given in Table 3.

As shown in Table 3, the DR and FPR of the Markov chain change with the sliding window size. Apparently, the support number of the abnormal point within the sliding window size affects the detection result. Hence, we selected the size of the sliding window size, 10, and set the testing support number from 4 to 8 to estimate the performance of sliding window fusion, where the testing telemetry series is the solar temperature. The detection results are given in Table 4.

As shown in Table 4, the FPR with different support numbers ranges from 0% to 6.6%. In real application, the support number is impossible to set effectively in advance. In other words, it may cause the fluctuation of DR and FPR. As a comparison, the Markov chain is not required to set this parameter that can increase the robustness of the detection model.

### 5.3. Case Study: Experiments on Telemetry Series with Anomalies

**Figure 15:**
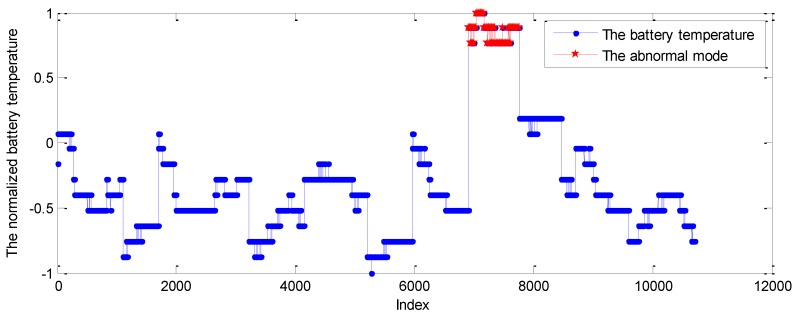
The battery temperature series.

In this part, the telemetry series of battery temperature from a spacecraft was applied to test the performance of the proposed method in the real application. Under normal circumstances, the battery temperature series change within limits. The testing temperature series were from 8th April to 13th April, while the points of 12th April beyond the normal range were labeled based on expert experience. The probability prediction method is the GPR model. The training data samples from 9th April were applied to train the GPR model. In addition, the normal telemetry points from 10th April were used to train the Markov chain. Then, the telemetry series from 11th April to 13th April were set as the testing series. The battery temperature series is given in Figure 15.

As shown in Figure 15, the battery temperature series has no evident period. Conversely, this telemetry is dynamic at the normal limitation. When the temperature value exceeds the normal range, it will be controlled by the telecontrol command to make the values get back to the normal range.

**Figure 16:**
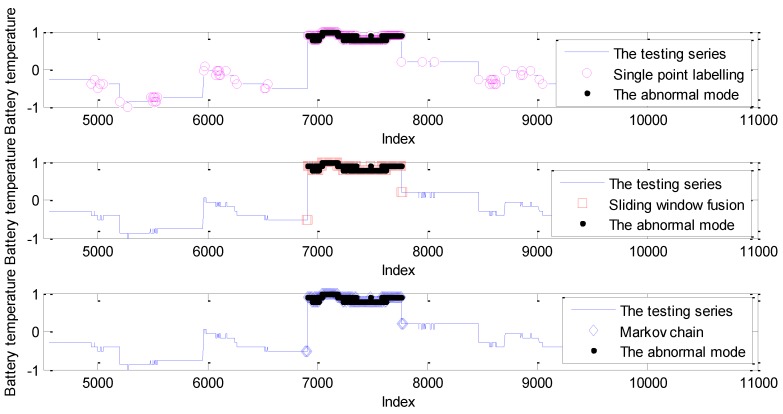
Anomaly detection with different labelling strategies.

The embedding dimension of the GPR model is 37, which was determined by autocorrelation analysis. Given the high sampling rate, the sliding window size was set to be 20 to cover 10 seconds of points. The detection results based on the probability prediction model with three labelling strategies are given in Figure 16.

As shown in Figure 16, some abrupt points were labeled mistakenly which cannot be modeled by the probability prediction model. As a comparison, the detection with the Markov chain can decrease these isolated false alarms. The quantified detection results are shown in Table 5.

As shown in Table 5, all the detection strategies can identify these abnormal points; the main reason is that these points are labeled by the setting fixed threshold in the aerospace area. The values of these points are larger than the normal range. Thus, detection with probability prediction can help to identify them well. Moreover, the labelling strategy of sliding window fusion and the Markov chain can reduce the isolated false alarms. In particular, false positives with sliding window fusion are better than with the other two strategies. The conclusion is similar to that of the experiments on the simulated data sets. If all the points can be detected with the original prediction, the advantage of the Markov chain is weakened. However, the detection of the Markov chain has no relation with the support number of points in the sliding window size, whose robustness is better than that of sliding window fusion.

## 6. Conclusions

In this work, a fusion model of anomaly detection with probability prediction and the Markov chain model was proposed to mitigate the isolated false alarms and improve the detection rate for collective anomalies. Firstly, compared with the single labelling strategy, the Markov chain model was trained with the sliding window to label the whole subseries. The introduction of sliding window to Markov chain can decrease the isolated false positives in telemetry series. Furthermore, given the independent assumption of the points within the testing sliding window, the sliding window fusion labelling cannot model the abnormal mode formed by points. The proposed fusion method can compute the transmission probability of the testing sliding window, which improves the detection rate for the collective anomalies. The experiments on the simulated data sets verified the performance improvement on the isolated false alarms and detection rate for the collective anomalies. In particular, the testing on the normal telemetry series and the abnormal telemetry showed the real applicability for anomaly detection of the telemetry.

There is also some work that needs to be conducted in the future. (1) The original series is processed to a binary sequence with probability prediction that should be further refined to different states to improve the learning ability of the Markov chain. (2) Now the maximum negative logarithmic value of support probability is set as the threshold for the testing series that has high requirements on the validation data, the threshold should be designed to be the soft threshold with more experiments. (3) The sliding window size has a great influence on the anomaly detection result that should be optimized in the next work.

## Figures and Tables

**Table 1:** The detection results with three labelling strategies under different sliding window sizes.

Data/Model	Strategy	Indices	5	6	7	8	9	10
Keogh Data/GPR model	Single point	DR	36.36% 1.50%	36.36% 1.50%	36.36% 1.50%	36.36% 1.50%	36.36% 1.50%	36.36% 1.50%
		FPR						
	Sliding window	DR	51.52% **1.50%**	24.24% **0.45%**	51.52% **0.60%**	30.30% **0.60%**	60.61% **0.75%**	30.30% **0.75%**
		FPR						
	Markov chain	DR	**84.85%**	**90.91%**	**96.97%**	**100.00%**	**100.00%**	**100.00%**
		FPR	3.75%	4.65%	5.25%	5.85%	6.45%	7.05%
Keogh Data/RVM model	Single point	DR	30.30% 5.85%	36.36% **1.50%**	36.36% **1.50%**	36.36% **1.50%**	36.36% **1.50%**	36.36% **1.50%**
		FPR						
	Sliding window	DR	15.15% **1.95%**	30.30% 2.70%	30.30% 1.50%	33.33% 2.25%	30.30% 1.65%	33.33% 2.55%
		FPR						
	Markov chain	DR	**63.64%**	**69.70%**	**75.76%**	**84.85%**	**87.88%**	**93.94%**
		FPR	4.20%	6.00%	6.90%	7.80%	9.60%	11.09%
Ma Data/GPR model	Single point	DR	48.48% 1.65%	48.48% 1.65%	48.48% 1.65%	48.48% 1.65%	48.48% 1.65%	48.48% 1.65%
		FPR						
	Sliding window	DR	63.64% **1.20%**	57.58% **0.00%**	72.73% **1.20%**	48.48% **0.00%**	51.52% **0.15%**	51.52% **0.15%**
		FPR						
	Markov chain	DR	**100.00%**	**100.00%**	**100.00%**	**100.00%**	**100.00%**	**100.00%**
		FPR	4.05%	4.95%	5.25%	5.55%	5.85%	6.15%
Ma Data/RVM model	Single point	DR	66.70% 6.30%	66.70% 6.30%	66.70% 6.30%	66.70% 6.30%	66.70% 6.30%	66.70% 6.30%
		FPR						
	Sliding window	DR	81.82% 0.90%	90.91% **2.25%**	90.91% **0.60%**	96.97% **2.25%**	96.97% **0.75%**	**100.00%** **1.05%**
		FPR						
	Markov chain	DR	**84.85%**	**90.91%**	**96.97%**	**100.00%**	**100.00%**	**100.00%**
		FPR	3.00%	4.20%	4.95%	5.55%	4.95%	5.55%

**Table 2:** The true skill statistic (TSS) with the Markov chain under different sliding window sizes.

Data	Model	5	6	7	8	9	10
Keogh Data	GPR model	81.10%	86.26%	91.72%	**94.15%**	93.55%	92.95%
	RVM model	59.44%	63.70%	68.86%	77.05%	78.28%	**82.85%**
Ma Data	GPR model	**95.95%**	95.05%	94.75%	94.45%	94.15%	93.85%
	RVM model	81.85%	86.71%	92.02%	94.45%	**95.05%**	94.45%

**Table 3:** The quantitative detection results for different series with the GPR model.

Data	Strategy	5	6	7	8	9	10
Solar voltage	Single point	4.80%	4.80%	4.80%	4.80%	4.80%	4.80%
	Sliding window	2.40%	**1.40%**	**1.80%**	**0.00%**	**0.00%**	**0.00%**
	Markov chain	**1.00%**	2.60%	4.80%	3.60%	2.00%	3.40%
Solar current	Single point	2.80%	2.80%	2.80%	2.80%	2.80%	2.80%
	Sliding window	**1.00%**	**0.00%**	**0.00%**	**0.00%**	**0.00%**	**0.00%**
	Markov chain	2.20%	4.20%	5.40%	5.60%	2.60%	3.00%
Battery voltage	Single point	3.40%	3.40%	3.40%	3.40%	3.40%	3.40%
	Sliding window	3.20%	1.80%	2.22%	2.22%	2.60%	**0.00%**
	Markov chain	**0.00%**	**0.00%**	**1.40%**	**1.80%**	**2.20%**	2.60%
Solar temperature	Single point	**3.80%**	3.80%	**3.80%**	3.80%	3.80%	3.80%
	Sliding window	6.00%	**3.40%**	4.20%	**2.20%**	**2.60%**	**0.00%**
	Markov chain	4.00%	5.00%	6.20%	7.40%	5.40%	0.00%

**Table 4:** The detection of false positive rate (FPR) with sliding window fusion under different support numbers.

Telemetry Series	4	5	6	7	8
Solar voltage	6.60%	3.00%	0.00%	0.00%	0.00%

**Table 5:** The detection results based on the GPR model under different labelling strategies.

Data	Strategy	FPR	DR
Battery temperature	Single point labelling	1.45%	100.00%
	Sliding window Fusion	0.42%	100.00%
	Markov chain	0.75%	100.00%
